# Taste and mouthfeel assessment of porous and non-porous silicon microparticles

**DOI:** 10.1186/1556-276X-7-407

**Published:** 2012-07-20

**Authors:** Qurrat Shabir, Cyrus Skaria, Heather O Brien, Armando Loni, Christian Barnett, Leigh Canham

**Affiliations:** 1 pSiMedica Limited, Malvern Hills Science Park, Malvern, Worcestershire, WR14 3SZ, UK; 2 Zentiva Group, k.s. (Part of the Sanofi Group), U Kabelovny 130, Prague 10, 102 37, Czech Republic

**Keywords:** Porous silicon, Silicic acid, Mouthfeel, Organoleptic, Taste, Food, Triangle test

## Abstract

Unlike the trace minerals iron, copper and zinc, the semiconductor silicon has not had its organoleptic properties assessed. Nanostructured silicon provides the nutrient orthosilicic acid through hydrolysis in the gastrointestinal tract and is a candidate for oral silicon supplements. Mesoporous silicon, a nanostructured material, is being assessed for both oral drug and nutrient delivery. Here we use taste panels to determine the taste threshold and taste descriptors of both solid and mesoporous silicon in water and chewing gum base.

Comparisons are made with a metal salt (copper sulphate) and porous silica. We believe such data will provide useful benchmarks for likely consumer acceptability of silicon supplemented foodstuffs and beverages.

## Background

Ever since the first silicon deprivation studies on animals [1,2], evidence has slowly accumulated that dietary silicon is beneficial to bone and connective tissue health [3]; and yet, the exact biological role(s) remain(s) an enigma. The essentiality of silicon for mammals, and humans in particular, remains questionable [4], and there is therefore no current recommended daily allowance. The paucity of data with regard to randomised, double-blind, and placebo controlled human studies to date has also meant that various health claims have not been substantiated by regulatory authorities [4]. Nonetheless, there is increasing scientific interest and scrutiny of the potential nutritional functions of silicon-based compounds and even medically biodegradable forms of pure elemental solid silicon [5].

There are also ongoing studies and proponents for oral silicon supplementation [6,7]. Mesoporous silicon is a very high surface area form of silicon that is biodegradable within the human body [8,9] and can be loaded with drugs or nutrients [5,10,11]. Its biodegradability is a consequence of nanostructuring, the mesoporous forms having high concentrations of pores ranging from 2 to 50 nm diameter. The resultant nanoscale silicon (Si) skeleton has markedly different properties to those of solid silicon [5,8-10].

Here we use taste panels to determine the taste thresholds and descriptors for both solid silica (Si), porous silica (pSiO_2_) and oxidised mesoporous silicon (OpSi) microparticles. We use drinking water as a control carrier liquid for beverages and chewing gum base as a control for solid foodstuffs. We compare the taste of different materials of similar particle size and the mouthfeel of a given material at varying particle sizes.

## Methods

Mouthfeel is sensitive to microparticle size distribution so this was measured for each powder type by Malvern Mastersizer 2000 (Malvern Instruments Ltd., Malvern Worcestershire, UK) using sonication in water. Table 1 lists the silicon-based powder materials assessed in this study.

**Table 1:** Silicon-based powders/material used in the study

**Silicon-based powder**	**Details**	**D10 (μm)**	**D50 (μm)**	**D90 (μm)**	**Pore volume (ml/g)**	**Shape**
Solid silicon (Si)	Metallurgical grade (Sigma-Aldrich Corporation, St. Louis, MO, USA – 99% purity)	4	12	27	0	Acicular
Solid Silicon (Si)	Metallurgical grade (Elkem AS Silicon materials. Norway, >99%)	2	4	9	0	Acicular
Oxidised porous silicon (OpSi)	Oxidation of milled powder (from anodised membrane) at 600°C (pSiMedica Ltd., Malvern, Worcestershire, UK)	0.4	4	12	0.55	Acicular
Oxidised porous silicon (OpSi)	Oxidation of milled powder (from anodised membrane) at 600°C (pSiMedica Ltd).	3	23	120	0.55	Acicular
Porous silica (pSiO_2_)	Silica gel (Silicycle S100007B, Silicycle Inc. Quebec City, Quebec, Canada)	3	11	18	0.78	Spherical
Porous silica (pSiO_2_)	Silica gel (Davisil LC250)	85	134	194	1.85	Acicular

Free-standing porous silicon membranes were fabricated by electrochemical anodisation of 6 inch diameter p + type wafers. The porous silicon flakes were rotor milled to obtain a powder with two different particle size distributions (D50 values of 4 and 23 μm). These porous silicon powders were subsequently oxidised in the air under static conditions at 600°C for 15 min. Solid silicon powders of two different size distributions (D50 values of 4 and 12 μm) were used as received from the manufacturer, as were the two porous silica powders with D50 values of 11 and 134 μm.

Gas adsorption/desorption analysis was carried out using a Micrometrics TriStar instrument (Micrometrics UK Ltd., Bedfordshire, England) with sample degassing at 70°C under vacuum for 1 hour and Brunauer-Emmett-Teller/Barrett-Joyner-Halenda (BET/BJH) analysis of the isotherms [12]. Drying under vacuum was found to lower both the degas temperature and time required, compared to the flowing nitrogen treatments. The efficacy of the degassing protocol was confirmed by analysis of Davisil LC250 (Sigma-Aldrich Corporation, St. Louis, MO, USA) for which the surface area, pore volume and mean pore diameter data repeatedly agreed with the manufacturer’s specification.

### Tasting of silicon powders/salts in water

The various silicon powders listed in Table 1 were dissolved/dispersed in drinking water (The National Forest Vending Co. Ltd., Newthorpe, Nottingham, UK, pH 6.8 at source) at different concentrations. The taste solutions of metallic salts (ultrapure grade) and silicon-based powders were prepared in drinking water according to International Standards Organization guidelines 3972:1991 using the purest salts available and the powders listed in Table 1.

The screening tests were first performed with the purpose of defining individual threshold concentrations that can be detected. Volunteers were presented with cups coded with random numbers; each cup contained a 20 ml test solution (in varying concentrations). When a subject perceived a distinct taste sensation, he or she recorded and described the particular taste. Once the individual’s threshold was defined, in the next session the subject underwent a modified triangle test. The triangle test is a three-product test in which the task is to identify one sample that differs from the other two. Three containers of identical volume, covered with aluminium foil, were presented to volunteers and they were asked to identify the odd sample and indicate the perceived taste. The samples were coded in a random order (e.g. CCA, ABA and ABC) with one sample being the test and the other two being pure water. The major impurities of the drinking water are listed in Table 2.

**Table 2:** Drinking water composition

**Ions present in drinking water**	**Ca**	**Mg**	**K**	**Na**	**Cl**^**-**^	**SO**_**4**_^**2-**^	**NO**_**3**_**-**	**Fe**
Levels (microgram per millilitre)	95	39	6	15	45	132	12	0.08

### Preparation of chewing gum pellets

Si, OpSi and pSiO_2_-loaded chewing gum pellets were made by initially vortex mixing a 27 g batch of chewing gum base powder (Cafosa Gum Ltd., Barcelona, Spain) with 3 g of the test substance. Aliquots of 1 g were then cold pressed into pellets using a 5-mm die set with 10 kN force for 30 s.

### Chew-out test protocol

The chew-out tests for metallurgical grade silicon and mesoporous silicon particles were done by a 10-person panel. The volunteers were asked to chew the pellet for 2 min. Two minute chews were followed by the collection of the saliva and rinse water for analysis of loss of silicon particles during chewing and recording the observations. The volunteers were asked to grade the samples for grittiness, taste and aftertaste.

### Release of silicic acid into water and its tasting

Chew-out tests were also mimicked *in vitro* by mechanically grinding pellets in Tris buffer at pH 6.8/artificial saliva for 0, 2 and 10 min. The release of silicic acid in media was measured at different time points and compared with silicic acid release from pellets without grinding. The media were filtered and analysed for silicic acid content.

The taste of a silicic acid solution of 58 μg/ml in deionized water was also judged using a triangle test. Mesoporous silica (Davisil, pore diameter 15 nm, particle size 52.5 μm (D50)) was completely dissolved in Tris buffer over a 12-day period. The samples from this solution were analysed for determination of the silicic acid released in the media. From the same sample, 6 ml aliquots were presented to volunteers in sets of three, where only one sample was the test and other two were deionised water used in preparation of buffer. The volunteers underwent the triangle test again as described earlier.

### Silicic acid assay

The silicic acid content was measured by spectrophotometry using molybdenum blue assay [13]. It is based on the reaction of silicic acid [Si(OH)_4_ with molybdic acid (or ammonium molybdate) at pH 1.5 to 2 to form the yellow isomer beta silicomolybdate (SiMO_12_O_40_)^4−^. This molybdate complex is then reduced by sodium disulphite to silicomolybdenum blue to increase spectrophotometric sensitivity. This allows determination of silicic acid concentration in the range of 10 to 70 μgs/ml with a variance of ±10%.

## Results and discussion

### Results

The mouthfeel of particles is very important in food products as it affects mastication and overall taste sensation of the product. In our study we chose particles (silicon/silica) of varied sizes to study the grittiness and any possible metallic taste or aftertaste of chewing gum pellets. All the particles used were analysed for particle size distribution and BET analysis for measuring porosity or pore volume.

**Figure 1:**
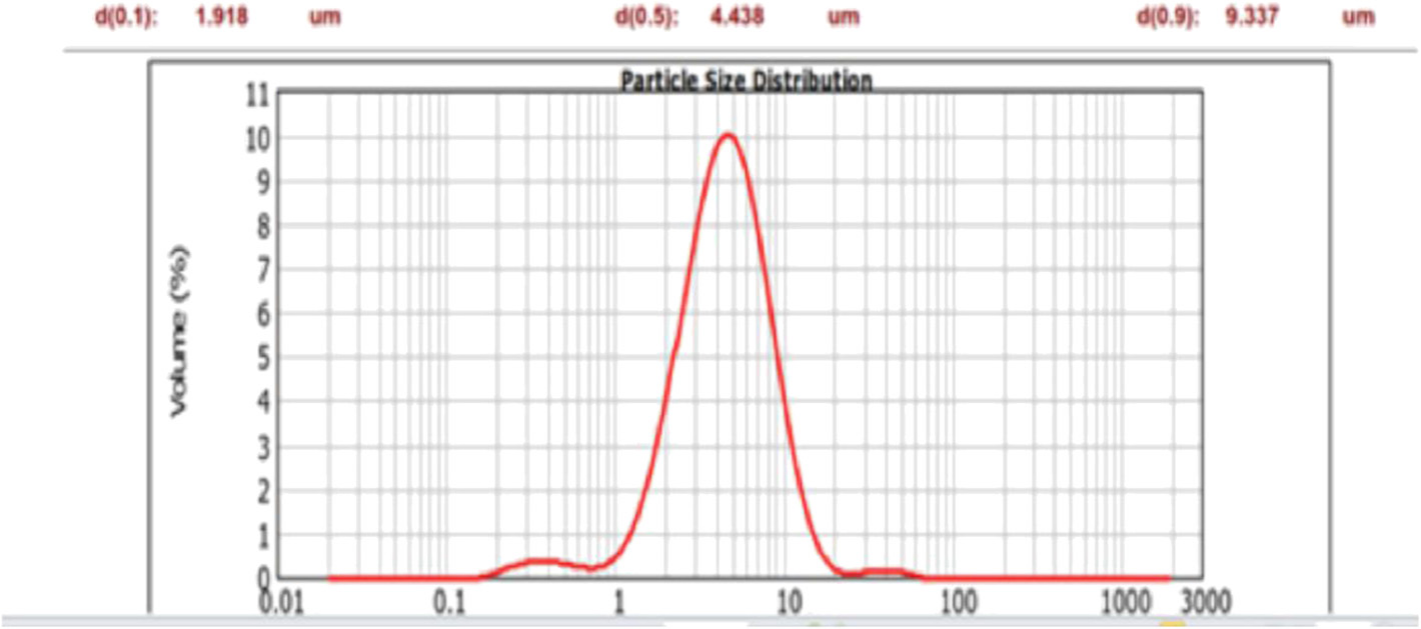
Particle size distribution of solid silicon particles by Malvern Mastersizer.

**Figure 2:**
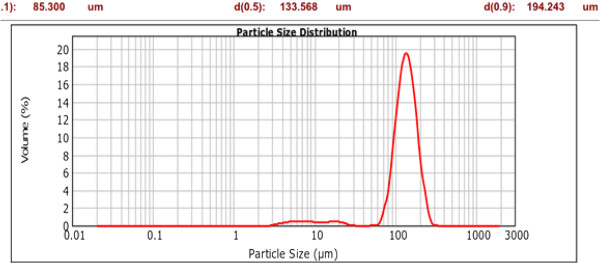
Particle size distribution of Davisil particles by Malvern Mastersizer.

Figures 1 and 2 illustrate the particle size distribution of two divergent samples from the six different particle sizes used in the study. Solid silicon powder with the smallest particles had a (D50) particle size of 4.4 μm whereas the largest mesoporous silica particles had a mean (D50) particle size of 134 μm.

The taste of different types of silica/silicon powder was compared by using batches of similar particle size; namely, solid silicon (D50 of 4 μm), OpSi powders (D50 of 4 μm) and pSiO_2_ particles of (D50 of 11 μm). The data shown in Table 3 indicate the concentrations of solutions which the panel could detect significantly when compared with pure drinking water. Almost 98% of volunteers could detect copper sulphate and solid silicon at 0.2 and 10 mg/ml respectively; therefore, the level of significance of the test was the highest (*p* < 0.001). Oxidized porous silicon could be tasted at 1 mg/ml by 85% of the panel (*p* < 0.01), whereas only 58% of the panel could detect porous silica in water at the same concentration and therefore, the level of significance for the latter is only *p* < 0.2.

**Table 3:** Statistical significance of identifying silicon-based powders in water with triangle test

**Test material in water**	**Concentration (mg/ml)**	***p*- value**
Copper sulphate	0.2	0.001
Solid silicon	1.0	0.001
Oxidised porous silicon	1.0	0.01
Porous silica	1	0.2

With regard to taste descriptors, ‘supertasters’ within the panel of 22 volunteers ascribed a ‘chalky’ taste to silica in water and ‘metallic taste’ to bulk silicon. Their taste descriptors for oxidized porous silicon were ‘no metallic taste’ or ‘no off-taste’.

**Figure 3:**
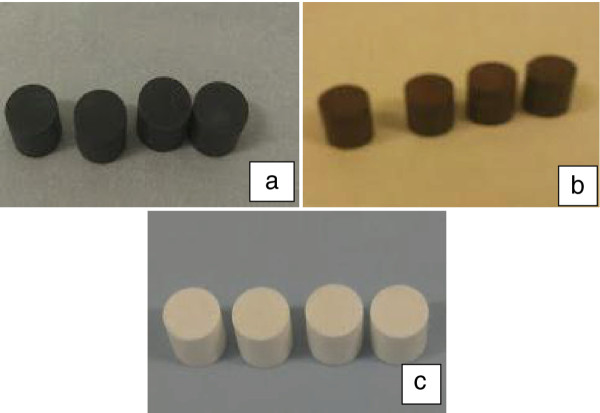
Chewing gum pellets made with (a) bulk, (b) mesoporous silicon and (c) silica powders blended with gum.

The pellets made from various silicon/silica materials are shown in Figure 3. Those made with solid silicon are black (Figure 3a), those with oxidised porous silicon are brown (Figure 3b) and those with porous silica were off-white (Figure 3c). Since we wanted to explore at which size the grittiness of silicon/silica particles in chewing gum pellets could be detected, a wide range of the particle size (4 to 134 μm) was chosen. The comparison of mouthfeel for bulk and porous silicon utilized similar particle size distribution.

The chewing pellets prepared all weighed 1 g and they were presented to the volunteers in a blind manner to avoid biased taste results. Chew-out tests from gum samples showed varying mouthfeel for different material groups. For 10 wt.% OpSi-loaded gum, it would appear that a D90 of <12 μm would be acceptable, and 54% of volunteers found no ‘off-taste’ or ‘aftertaste’ at this high loading. The 10 wt.% pSiO_2_ gums, prepared from either particle size distribution, consistently had a ‘chalky’ taste. Such a high loading of particles had a significant effect on the mechanical properties of the pellets for all the test samples.

**Figure 4:**
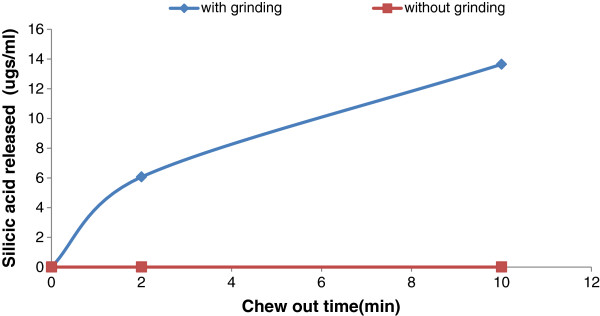
Silicic acid release from chewing pellets in artificial saliva/Tris buffer pH 6.8.

The *in vitro* study of biodegradation of the OpSi particles (D50 of 4 μm) in mechanically grounded gum (Figure 4) found a measurable level of silicic acid for storage times of 2 and 10 min. In contrast, pellets immersed in artificial saliva/Tris buffer pH 6.8 without grinding exhibited a negligible release of silicic acid (Figure 4), indicating the importance of mastication forces for accelerating the degradation of silicon/silica particles.

The threshold detection level for silicic acid in water was not quantified. Nevertheless, selected members of the panel were clearly able to identify highly concentrated silicic acid solutions from water controls with 100% accuracy. Their taste descriptors for the 58 μg/ml orthosilicic acid solution were ‘bitter sweet’ or ‘sweet’.

### Discussion

The primary objective of the study was to gauge the perceived taste of the semiconductor silicon, in solid and porous microparticulate form. The semiconducting silicon exposed to air or an aqueous environment will be covered in an ultrathin native oxide, so one might conclude that it is silica rather than silicon that is being assessed by the taste buds of the tongue. This becomes even more relevant for silicon structures that have been intentionally thermally oxidised (refer to Table 1).

However, there was a clear disparity between the panel perception of the taste of the solid silicon and the silica structures. This suggests that the ultrathin native oxide of silicon might not be effective in protecting taste bud exposure to the underlying silicon in the corrosive mouth environment. This is not too surprising, considering that much thicker plasma-enhanced chemical vapour deposition silica films on silicon were found to continuously corrode *in vivo*[14].

In the drinking water tests the particles are exposed to diluted human saliva for only a very short period of time (30 s). In the chew-out test they are exposed to both mastication forces and diluted human saliva for longer periods (2 min). Microparticle breakage exposes fresh surfaces to saliva and to the tongue. In addition, the biodegradability of mesoporous silicon is now established [8,9], so one would expect more degradation of the mechanically weaker porous silicon particles in the mouth, exposing material underneath that of the ultrathin oxide coating. Prior *in vitro* studies (QS and A Pokale, unpublished work) have shown that the rate of biodegradation is lowered as a result of thermal oxidation over the 300°C to 800°C temperature range. Significant silicic acid release still occurs after 600°C oxidation, even in the absence of mastication forces, but over many days of storage in simulated body fluids.

The data of Figure 4 (upper trace) support the statement that the oxidised porous silicon particles underwent significant biodegradation under the chew-out test conditions. However, the Figure 4 data (lower trace) also suggest that for the water test conditions, a very low degree of particle degradation occurred. It is also unclear as to whether the levels of silicic acid released into the mouth by either test would themselves have imparted a significant taste.

## Conclusions

Mesoporous silicon particles are being evaluated for their use in nutrient delivery and in oral care formulations like toothpaste and chewing gum. Taste and mouthfeel are very important factors in consumer acceptance of such products. This preliminary study demonstrates that semiconducting mesoporous silicon is likely to have relatively ‘bland’ taste, intermediate between those of the insulator silica, and the strong taste of metallic micronutrients like copper, zinc and iron. Detailed studies need to be conducted to assess the possible uses of porous silicon/silica in food products, but from an organoleptic perspective these materials would appear to be acceptable at moderate concentrations and microparticle size distributions. Further work is also required regarding establishing the detection threshold for orthosilicic acid.

## Competing interests

The authors declare that they have no competing interests.

## Authors' contributions

QS prepared samples and carried out the taste and mouthfeel panel studies, performed the statistical analysis, carried out degradation studies and helped in drafting the paper. CS prepared the gum pellets and initiated the study. HO conducted particle size analysis. AL manufactured the porous silicon powders and amended/reviewed the paper. CB reviewed and amended the protocols and manuscript. LC conceived, designed and coordinated the study, and also drafted the paper. All authors have read and approved the final manuscript.
